# Acute normobaric hypoxia does not affect the simultaneous exercise-induced increase in circulating BDNF and GDNF in young healthy men: A feasibility study

**DOI:** 10.1371/journal.pone.0224207

**Published:** 2019-10-23

**Authors:** Zofia Piotrowicz, Małgorzata Chalimoniuk, Kamila Płoszczyca K, Miłosz Czuba, Józef Langfort

**Affiliations:** 1 Institute of Sport Sciences, The Jerzy Kukuczka Academy of Physical Education, Katowice, Poland; 2 Department of Tourism and Health in Biała Podlaska, The Józef Piłsudski University of Physical Education, Warsaw, Poland; 3 Department of Kinesiology, Institute of Sport, Warsaw, Poland; 4 Department of Sports Theory, The Jerzy Kukuczka Academy of Physical Education, Katowice, Poland; University of Bourgogne France Comté, FRANCE

## Abstract

Physical exercise has a neuromodulatory effect on the central nervous system (CNS) partially by modifying expression of neuropeptides produced and secreted by neurons and glial cells, among which the best examined are brain-derived neurotrophic factor (BDNF) and glial cell line-derived neurotrophic factor (GDNF). Because both neurotrophins can cross the brain-blood barrier (BBB), their blood levels indirectly reflect their production in the CNS. Moreover, both neuropeptides are involved in modulation of dopaminergic and serotoninergic system function. Because limited information is available on the effects of exercise to volition exhaustion and acute hypoxia on CNS, BDNF and GDNF formation, the aims of the present study were to verify whether 1) acute exercise to exhaustion in addition to neurons also activates glial cells and 2) additional exposure to acute normobaric moderate hypoxia affects their function. In this feasibility study we measured blood concentrations of BDNF, GDNF, and neuropeptides considered as biomarkers of brain damage (bFGF, NGF, S100B, GFAP) in seven sedentary healthy young men who performed a graded exercise test to volitional exhaustion on a cycle ergometer under normoxic (N) and hypoxic conditions: 2,000 m (H2; FiO_2_ = 16.6%) and 3,000 m altitude (H3; FiO_2_ = 14.7%). In all conditions serum concentrations of both BDNF and GDNF increased immediately after cessation of exercise (p<0.01). There was no effect of condition or interaction (condition x time of measurement) and exercise on any of the brain damage biomarkers: bFGF, NGF, S100B, GFAP. Moreover, in N (0<0.01) and H3 (p<0.05) exercise caused elevated serum 5-HT concentration. The results suggest that a graded effort to volitional exhaustion in normoxia, as well as hypoxia, simultaneously activates both neurons and astrocytes. Considering that s100B, GFAP, bFGF, and NGF (produced mainly by astrocytes) are markers of brain damage, it can be assumed that a maximum effort in both conditions is safe for the CNS.

## Introduction

Physical effort stimulates many processes in the central nervous system (CNS), among which the best recognized are neurogenesis, brain synaptogenesis, synthesis and use of neurotransmitters, angiogenesis and increase of the blood flow in several cortical and subcortical areas [1–6]. Research from recent years revealed that beneficial changes causing an impact on brain structure and function are at least partly linked to neurotrophic factors produced and secreted by both neurons and glial cells. Among them, brain-derived neurotrophic factor (BDNF) is the best studied in terms of its effect on the CNS, whereas glial cell line-derived neurotrophic factor (GDNF) is another neurotrophin that is the subject of the latest research. These two neurotrophic factors belong to different families: BDNF to the family of neurotrophins and GDNF to the family of transforming growth factors-α (TGF-α) [7–9]. It is well documented that both acute and chronic exercise is a potent stimulus for increasing BDNF production by neurons in healthy adults [10,11]. Moreover, data show that along with elevated expression of BDNF in the brain there is a simultaneous increase of its blood levels in response to physical exercise [12–14]. This phenomenon may be explained by the fact that BDNF can cross the blood-brain barrier (BBB) in a bi-directional manner according to a concentration gradient [15]. It was also shown that the levels of GDNF increased during exercise in the spinal cord of young and old rats [16]. Glial cell line-derived neurotrophic factor can protect dopamine (DA) neurons against cell death in vitro [17]. It was shown that GDNF-induced increases in DA function are associated with a sustained increase in tyrosine hydroxylase (TH) phosphorylation at Ser31, which may be maintained for at least one month following its single striatal administration [18]. These results obtained on animals provide a support for the protective neurobiological influence of GDNF against motor deficits.

A single bout of intense exercise involving large muscle groups causes an increase in expression of BDNF in neurons. Brain-derived neurotrophic factor acts on tropomyosin (tyrosine) receptor kinase B (TrkB) expressing neurons [19,20] located near or far away from the cell bodies of its production to induce a cascade of events that regulate their energy balance [21] and promote functional and structural plasticity of the brain [22,23]. Among other effects, the binding of BDNF to TrkB triggers the transcription of the TH gene responsible for the conversion of tyrosine to dihydroxyphenylalanine (L-DOPA), which is a precursor of DA [24**–**26]. Furthermore, studies performed *in vitro* and *in vivo* revealed that the physiological role of BDNF was more closely functionally connected with the serotonergic system of the brain as compared with the dopaminergic system [9,17]. Importantly, the rate limiting enzyme of serotonin (5-HT) production, tryptophan hydrolase, requires the presence of oxygen as well as acetylcholine [27]. The commonly accepted viewpoint is that both aforementioned systems play a pivotal role in regulation of human movement [28,29]. For example, studies have shown that increasing the level of 5-HT can inhibit DA neurotransmission, which as a consequence might lead to a decrease in the activity of the motor cortex [30]. Moreover, accumulation of 5-HT in brain is considered the cause of central fatigue (CF), which may be the reason for cessation of intense, endurance physical effort involving large muscle groups [31].

Glia cells (astrocytes and microglia) are significantly responsible for the physiological state of the CNS. They participate in neurotransmission, support synaptogenesis and regulate concentrations of ions, neurotransmitters and neurohormones [32–34]. Astrocytes affect neural metabolism to supply neurons with an energy substrate, regulate concentrations of ions, and take part in neurotransmission [35]. Their role is particularly important in sustaining neuronal activity of neurons with decreased glycogen concentration by accelerating astrocyte-neurons lactate shuttle [36,37]. After brain injury, neuron regeneration is regulated by trophic factors released from astrocytes [38]. Astrocytes maintain the survival of neurons under ischemic conditions [39]. They are an element of BBB and thereby control the metabolic status of the CNS by changing its permeability [40]. In turn, microglia (together with astrocytes) constitute the immune system of the nervous system [41]. Both astrocytes and microglia are able to produce neurotrophins, which can cross the BBB, and can be measured in the blood; they include glial fibrillary acidic protein (GFAP), nerve growth factor (NGF), basic fibroblast growth factor (bFGF) and calcium binding protein B (S100B), but their formation and release during exercise and/or hypoxia is less recognized.

In addition to the positive effect on human health [42], physical exercise is one of the important factors modifying the state of the CNS. In normoxia, prefrontal cortex oxygenation during exhaustive exercise decreases as a result of an imbalance between a reduction of cerebral blood flow and elevated cerebral metabolic rate and O_2_ uptake [43]. Studies performed in vitro showed that the responsiveness of neurons to reduced O_2_ availability was immediate and changed their activity [44]. The impact of exercise on CNS function can be additionally affected by exercise–induced production of metabolic, humoral and molecular factors released from peripheral (systemic) tissues. Studies in humans in which overall brain energy metabolism was measured under acute hypoxia also showed that the energy state of the brain can be modified as indicated by increased glucose consumption and lactate production in humans [45].

The physiological effect of hypoxia on human body depends on many factors, among which the level of hypoxia, including low (equivalent to 500-1500m altitude), moderate (1500-3000m) and severe (>3000m), and time of exposure (acute vs. chronic lasting from several days to several weeks) seem to be the most important [46**–**48]. The best illustrative examples of the destructive effect of acute severe hypoxia are acute mountain sickness (AMS) and high-altitude headache [49]. Furthermore, during exposure to chronic hypoxia, the human organism has the ability to adapt to new conditions, but at the same time it is exposed to the debilitating effects of hypoxia [47]. In recent years, researchers have focused on the impact of Intermittent Hypoxia Exposure (short-term cyclic exposure to hypoxia; IHE) on human health, because there are indications that IHE may have a positive therapeutic effect on e.g. cardiac function in coronary patients or a favorable effect on the symptoms of bronchial asthma [50]. On the other hand, IHE in patients with obstructive sleep apnea is currently considered to be the potential major contributor to the cardiovascular, metabolic and neurocognitive complications associated with this disease [51]. Therefore, it seems that therapeutic vs. pathological effect of IHE depends on the dose (which consists of time exposure, severity of hypoxia, the number of cycles, etc.) [48]. Hypoxia is also used in the process of sports training to improve performance in normoxia [52–54]. One of the most popular conception is Intermittent Hypoxic Training (IHT) which involves staying in normoxia and training in hypoxic conditions. Also, in this case, it seems that the changes occurring as a result of IHT depend on the size of the hypoxic stimulus, but also on the intensity of the exercise performed under these conditions [55].

However, mechanism of hypoxia’s effect on the CNS is still poorly known. Some data suggest that exposure to hypoxia reduces cognitive functions and modifies central motor command [56,57]. The best illustrative examples of the destructive effect of hypoxia on the brain are acute mountain sickness (AMS) and high-altitude headache [49]. Hypoxia may cause CNS perturbation per se and/or secondary to alterations in neurotransmitter as well as hormonal/humoral factor levels. Therefore, the two aims of the present investigation were to verify: first, whether acute exercise to exhaustion in addition to neurons also activates glial cells (astrocytes and microglia), and second, whether additional exposure to acute normobaric moderate hypoxia affects their function. Considering the facts that intense physical exercise can modify the physiological state of neurons [10,11], and glial cells support the functioning of neurons, especially during reduced energy availability [35–37], we believe that acute exercise to exhaustion can activate astrocytes and microglia in addition to neurons. It is known that reduced oxygen availability can instantly change neuronal activity [44] and acute severe hypoxia can disrupt CNS function [49]. Moreover, during exhaustive exercise, oxygenation in the CNS decreases [43]. Therefore, we suppose that exposure to acute moderate hypoxia in combination with exercise to exhaustion can affects both neurons and glial cell functions. To test these working hypotheses, we measured selected serum neurotrophic factors produced by both neurons and glial cells (BDNF, GDNF, bFGF, NGF, S100B, GFAP) which can cross the BBB, and can be measured in the blood.

## Materials and methods

### Participants

This study examined seven healthy man, leading a sedentary lifestyle (n = 7; age range 19–22 years, mean age 20.1 ± 1.2 years; body mass 70.9 ± 12.9 kg; body height 175.4 ± 9.4 cm; fat content 12.4 ± 4.2%) who declared that they had not been in the last two years at an altitude of 1500m above sea level. Participants were recruited in November 2015. For three days before experiment, the subjects were on a standardized normocaloric (37kcal·kg^-1^·day^-1^) diet with 50–60% carbohydrate, 15–20% protein, and 20–30% fat. They were also obliged to abstain from strenuous exercise. No caffeine, tea, or alcohol was permitted during the 72 hours before the experiment. All subjects possessed a current medical examination, without any contraindications to performing exhaustive exercise in both normoxia and a hypoxic environment. The experimental procedures involved and the related risks was explained to all the participants verbally, and informed written consent was taken from each participant.

The research project was conducted according to the Helsinki Declaration and was approved by the Ethics Committee for Scientific Research at the Jerzy Kukuczka Academy of Physical Education in Katowice, Poland.

### Testing protocol

All of the participants performed a graded exercise test to volitional exhaustion (EVE) using the Excalibur Sport cycle ergometer (Lode BV, Netherlands) under normoxic (N) and hypoxia conditions, equivalent to 2,000 m altitude (H2; FiO_2_ = 16.6%) and 3,000 m altitude (H3; FiO_2_ = 14.7%). The graded exercise test began with a load of 40 W, with increments of 40 W every 3 minutes. The test was continued to exhaustion or until the participant was unable to maintain the minimal cadence of 60 rpm. If a participant terminated the test before completing a given workload, the maximum workload (WR_max_) was calculated from the formula WR_max_ = WR_k_ + (t/T x WR_p_) [58], where WR_k_ = previous workload, t = exercise duration with the workload until premature failure, T = duration of each workload, and WR_p_ = amount of workload by which exercise intensity increased during the test.

During the test, oxygen uptake (VO_2_), expired carbon dioxide (CO_2_) and minute ventilation (VE) were measured continuously using the MetaMax 3B telemetry spiroergometer (Cortex, Germany) in the breath-by-breath mode. The criteria of reaching VO_2max_ included: a plateau in the level of VO_2_ or a gradual decrease in peak VO_2_ during the maximal workload, respiratory exchange ratio (RER above 1.1), blood lactate concentration (LA above 8.0 mmol/l), as well as predicted maximal heart rate [59]. All participants finished the incremental test with the required criteria of reaching VO_2max_.

Normoxic and both hypoxic conditions were created using a normobaric chamber (LOSA HYP/HYOP-2/3NU system/LOWOXYGEN SYSTEMS, Germany) in the Hypoxia Laboratory of the Jerzy Kukuczka Academy of Physical Education. The order of conditions (N, H2, H3) in which the exercise test was carried out was a single-blind simple randomized controlled trial. The testing procedures in all trials were identical for all participants. The intervals between the trials were 7 days.

Before the first test, after an overnight fast, body mass, height and body composition of participants were determined using electrical impedance measurements (Inbody 720, Biospace Co., Japan). Two hours before each trial, participants ate a light breakfast (5 kcal/1 kg of body weight, 50% carbohydrates, 20% proteins, 30% fats). The tests in each trial were started with the collection of blood samples from the antecubital vein to determine resting biochemical selected variables: neuropeptides, DA, 5-HT and their metabolites. Additionally, capillary rest and post-exercise blood samples were used to determine LA concentration (Biosen C-line, EKF Diagnostics, Germany) and to determine acid-base equilibrium and capillary oxygen saturation of hemoglobin (SO_2capillary_) (RapidLab 248, Bayer Diagnostics, Germany). Blood was collected under the same conditions as the next test (i.e. N, H2, H3). Immediately after each test and 60 min after exercise, blood samples were again collected to determine DA, 5-HT and neuropeptides.

### Blood sampling

Blood samples from the antecubital vein were drawn using no anti-coagulant and processed for serum for the other biochemical assays. After 30 min, blood was centrifuged at 1500 x g for 15 min. The serum obtained was stored at -75°C until analyzed.

### Neurotrophic factors

Neurotrophins were measured by sandwich enzyme-linked-immunosorbent assay (ELISA) methods using a commercially available kits: Quantikine^®^ELISA kitR&D Systems (Minneapolis, USA) for BDNF; Biosensis (Thebarton, South Australia) for GDNF; EIAab Science Co. (Wuhan, China) for S100B, GFAP and bFGF; Biorbyt (Cambridge, UK) for NGF. All neuropeptides were measured according to the procedures supplied by the manufacturer. Next, the absorbance level was measured (POLARstar Omega Microplate Reader BMG Labtech GmbH, Germany, Ortenberg) and finally the total amount of neuropeptides in serum was converted into concentration based on a standard curve.

### Determination of DA and 5-HT by HPLC method

Dopamine, 5-HT and their metabolites: 3,4-dihydroxyl phenylacetic acid (DOPAC), homovanillic acid (HVA) and 5-hydroxyindoleacetic acid (5-HIAA), were assayed in the serum using high performance liquid chromatography (HPLC) with electrochemical detection Coulochem III model 520 (ESSA, Copenhagen, Denmark). Serum samples were mixed with 0.1M perchloric acid containing 22.5 ng/ml ascorbic acid (ASC, Sigma-Aldrich, St. Louis, MO, USA). After centrifugation at 15000g, 10 min, at 4°C, supernatant was filtered through a nylon syringe filter (Millipore, 0.22μm, CA, USA). Samples of 20 μl filtrate were injected into a high performance liquid chromatography system (Gynkotek, Copenhagen, Denmark) equipped with a Hypersil Gold (15 cm x 4.6 mm) column (Thermo Electron Corporation, Kleinostheim, Germany). The samples were eluted by a mobile phase made of 107 mM of Na_2_HPO_4_ x 2H_2_O, 107 mM citric acid, 0.3 mM octane-1 sulfonic acid sodium salt (OSA), 0.2 μM of EDTA, pH, 4.6, 1.5% methanol and 1.5% acetonitrile at a flow rate of 0.8 ml/min. The column temperature was set at 25°C. Peaks were detected by electrochemical detection (Coulochem III, ESSA, Copenhagen, Denmark) at potentials of E1 = -50 mV and E2 = 400 mV. Data were collected and analyzed using Chromeleon software run on a PC (Gynkotek, Copenhagen, Denmark). DA and 5-HT contents in the sample were calculated by extrapolating the peak area from a standard cure.

### Statistical analysis

The results of the study were analyzed using StatSoft Statistica 13.0 software. The results were presented as arithmetic means (x) and standard deviations (SD). The statistical significance was set at p<0.05. Prior to all statistical analyses, normality of the distribution of variables was checked using the Shapiro-Wilk test. The one-way analysis of variance (ANOVA) with repeated measures was used to determine the significance of differences in VO_2max_, WR_max_, ΔLA between three consecutive trials in different conditions. The intergroup differences between research trials (condition × time of measurement) in selected biochemical variables were determined using the two-way ANOVA for repeated measures. When significant differences were found, the post hoc Tukey’s test was used. Effect sizes (ESs) were calculated from standardized differences (Cohen’s d units). A-priori analysis (GPower 3.1 software) showed that for n = 7, while maintaining an acceptable power (1-β = 0.80) and α = 0.05, the test allows for detection of the effect size >0.80 (large) [60].

## Results

### Maximal workload and cardiorespiratory variables

Results of one-way ANOVA showed a significant (conditions) interaction for the WR_max_ (F = 5.08, p<0.05) and VO_2max_ (F = 7.25, p<0.01) during the incremental test in tested conditions (N, H2 and H3). Furthermore, SO_2capillary_ at rest (SO_2capillary_rest; F = 57.6, p<0.001) and immediately after the incremental test (SO_2capillary_max; F = 62.8, p<0.001) were also significantly different in all conditions.

**Fig 1:**
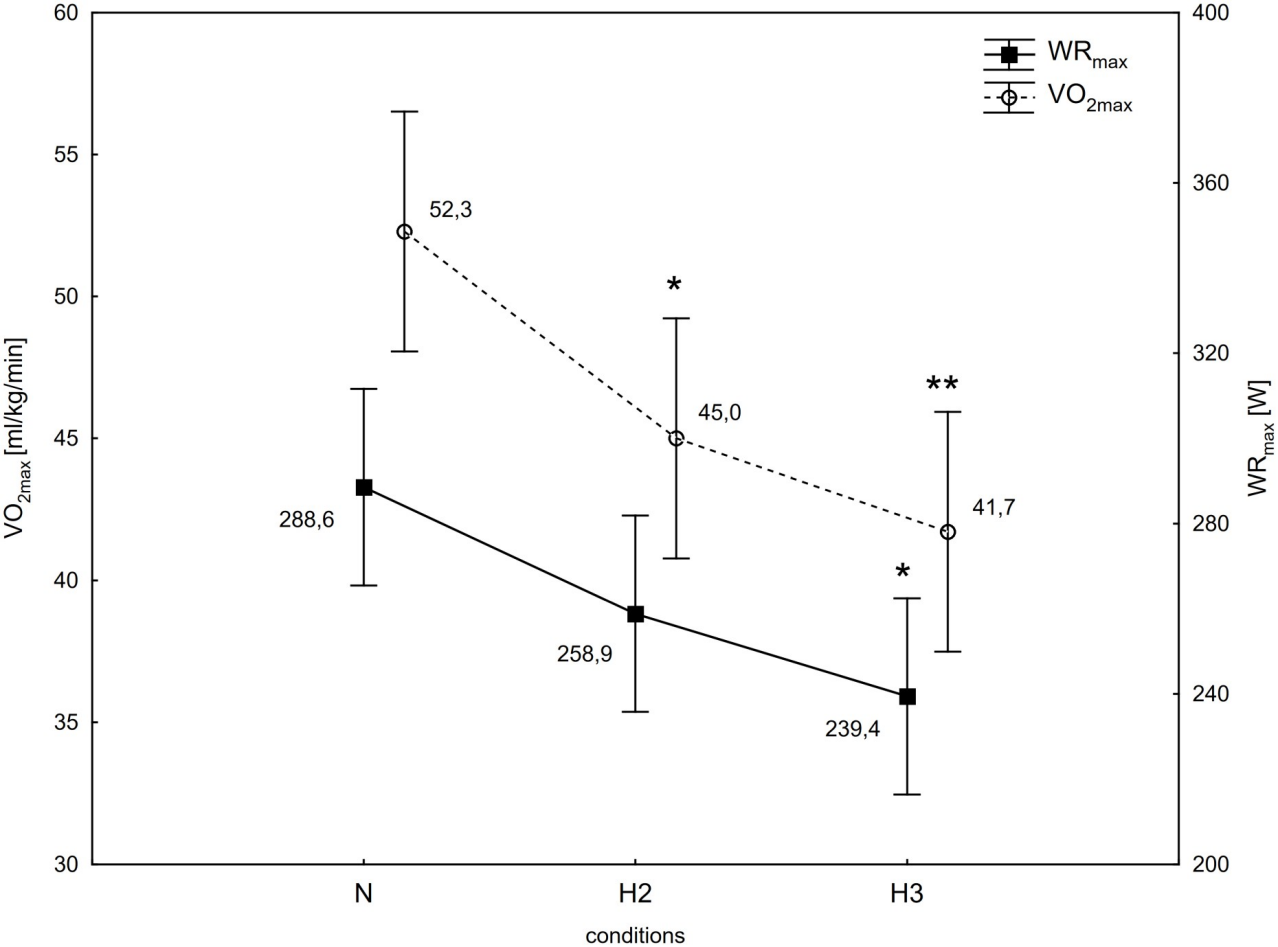
Maximal oxygen uptake (VO_2max_), and maximal workload (WR_max_) during incremental test performed in different conditions. N-normoxia, H2-hypoxia 2,000 m, H3-hypoxia 3,000 m; * p<0.05, ** p<0.01—statistically significant differences in relation to N.

**Fig 2:**
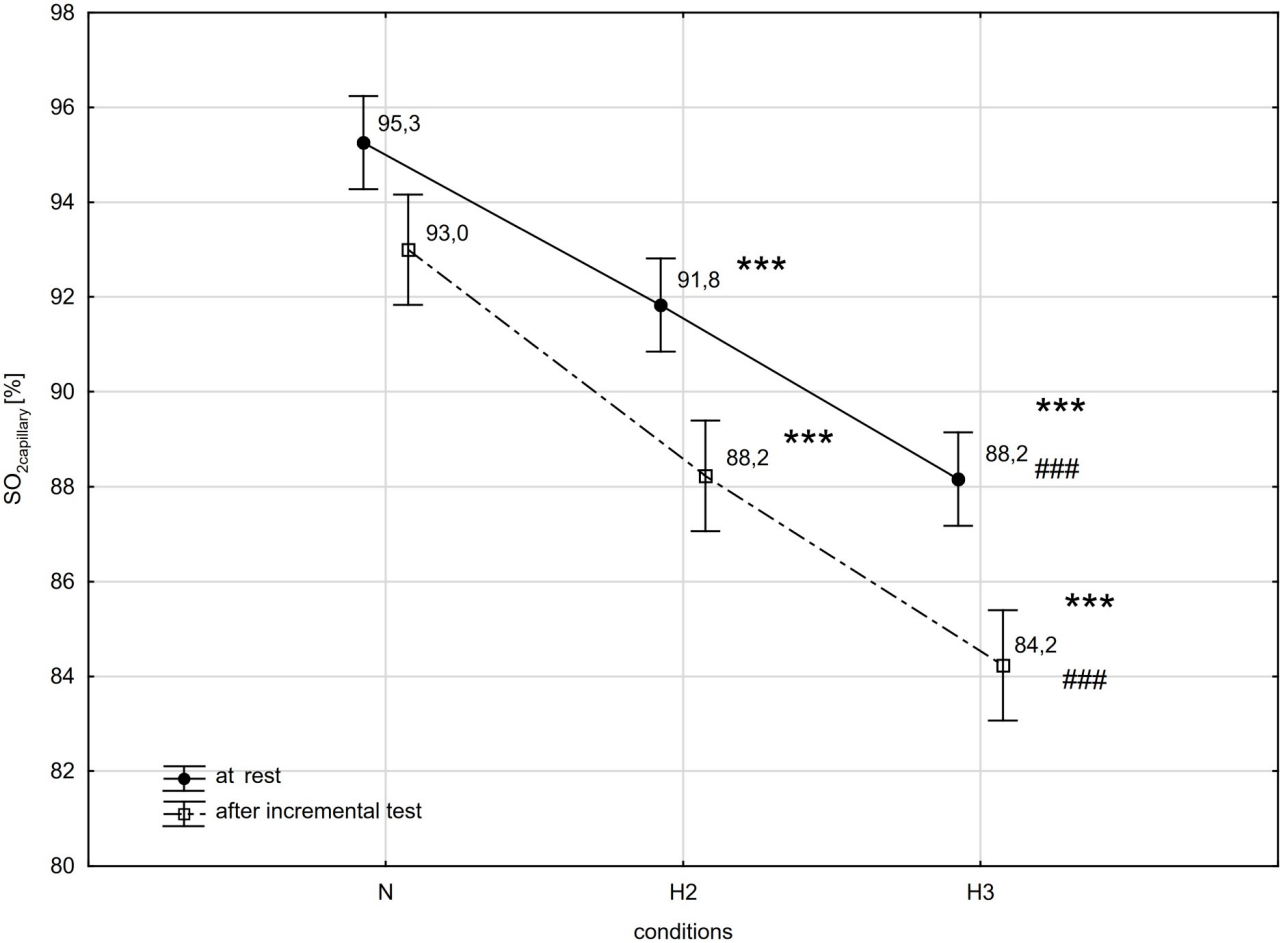
Decrease in capillary oxygen saturation of hemoglobin (SO_2capillary_) at rest and after incremental test in different conditions. N-normoxia, H2-hypoxia 2,000 m, H3-hypoxia 3,000 m; *** p<0.001—statistically significant differences in relation to N, ### p<0.001—statistically significant differences in relation to H2.

**Fig 3:**
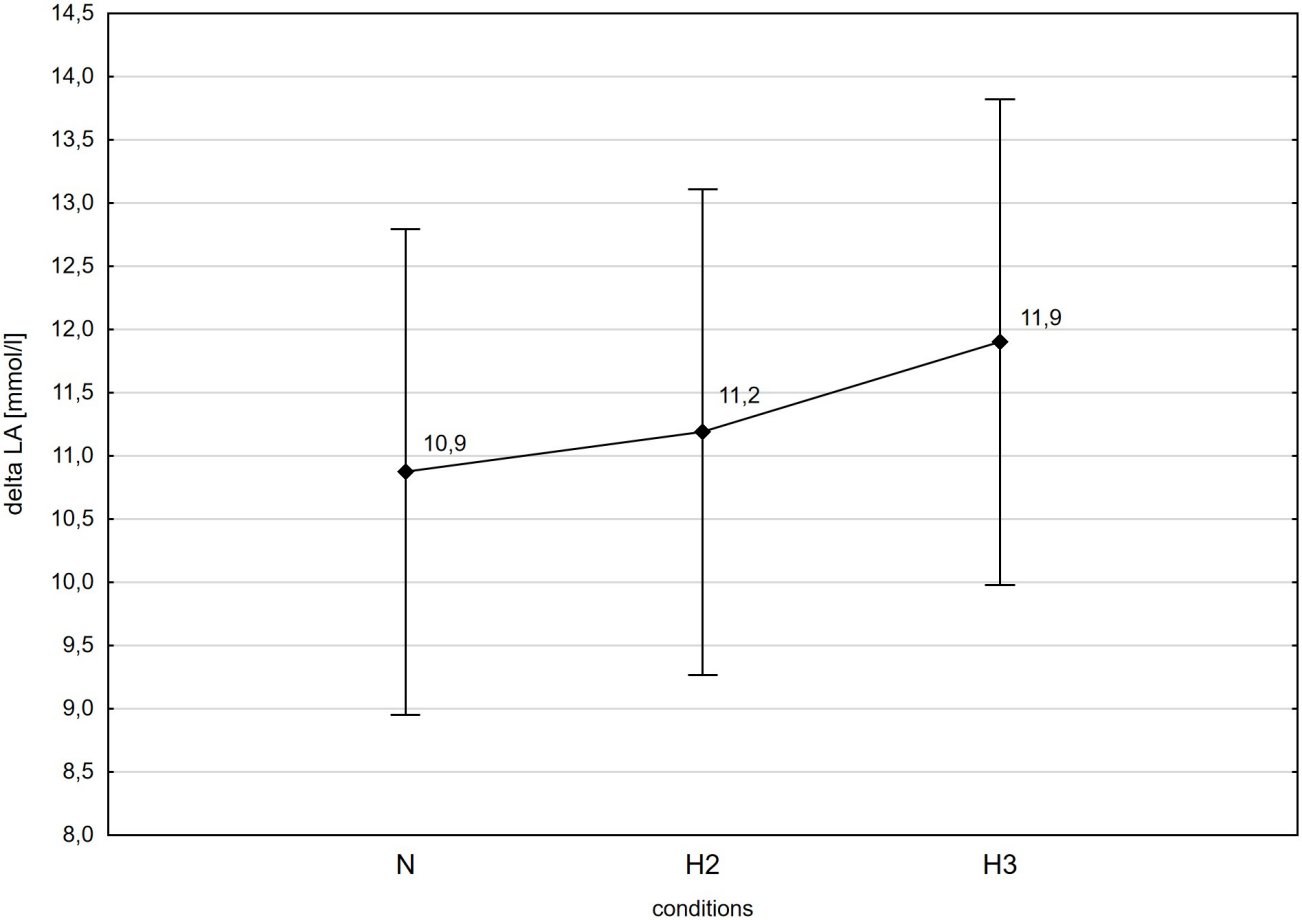
Increase in blood lactate concentration (LA_max_) during incremental test performed in different conditions. N-normoxia, H2-hypoxia 2,000 m, H3-hypoxia 3,000 m.

The post-hoc Tukey's test showed that WR_max_ decreased significantly (p<0.05; ES: 1.76) by 17% in H3 compared to the initial measurements in N (Fig 1). Although there was a decrease in WR_max_ in H2 compared to N, it was not statistically significant. Additionally, the values of VO_2max_ decreased significantly (p<0.05) in H2 and H3 compared to N respectively by 13.9 (ES: 1.56) and 20.2% (ES: 2.04; Fig 1). The SO_2capillary_rest, as well as SO_2capillary_max decreased significantly (p<0.001) in H2 (ES: SO_2capillary_rest—3.47; SO_2capillary_max—3.67) and H3 (ES: SO_2capillary_rest– 6.15; SO_2capillary_max– 5.58) compared to N. The similar significant (p<0.001) changes of this variables were also observed between H2 and H3 (ES: SO_2capillary_rest– 2.87; SO_2capillary_max—3.43; Fig 2). There were no statistically significant differences in delta values of the LA concentration in blood after the incremental test between N, H2 and H3 (Fig 3).

### Changes of neurotrophin factors

No significant interaction (condition x time of measure) effect was found for any of the measured neurotrophins (Table 1), but significant main effects of time of measurement (at rest, immediately after the test and 1 h recovery period) on BDNF (F = 25.45; p<0.001) and GDNF (F = 20.77; p<0.001) concentration were observed.

**Table 1:** Mean values of selected neurotrophins registered in the different conditions at rest, after incremental test (max) and after 1 h recovery period.

Variables	N			H2			H3		
	rest	max	after 1 h	rest	max	after 1 h	rest	max	after 1 h
**bFGF** [pg/ml]	3.22±0.87	5.06±1.09	2.50±0.70	3.39±1.20	3.32±2.13	4.01±2.30	3.96±1.96	5.25±2.25	3.86±1.90
**NGF** [pg/ml]	1313.1±517.3	1348.9±239.0	3016.6±2089.7	15101.4±17530.5	17023.1±22663.9	12654.3±14508.7	7290.±7224.7	8152.0±8250.0	8566.9±6320.6
**S100B** [pg/ml]	6.50±4.69	3.44±2.11	6.86±3.95	14.37±12.02	8.94±6.09	14.43±13.80	3.96±1.96	5.25±2.25	3.86±1.90
**GFAP** [pg/ml]	1.90±1.68	1.71±0.52	0.83±0.09	13.13±18.73	8.79±13.93	3.77±4.97	2.47±2.12	1.79±0.61	2.06±1.38

N- normoxia, H2—hypoxia 2,000 m, H3—hypoxia 3,000 m, BDNF—brain-derived neurotrophic factor, GDNF—glial cell line-derived neurotrophic factor, bFGF—basic fibroblast growth factor, S100B—calcium binding protein B, GFAP—glial fibrillary acidic protein

**Fig 4:**
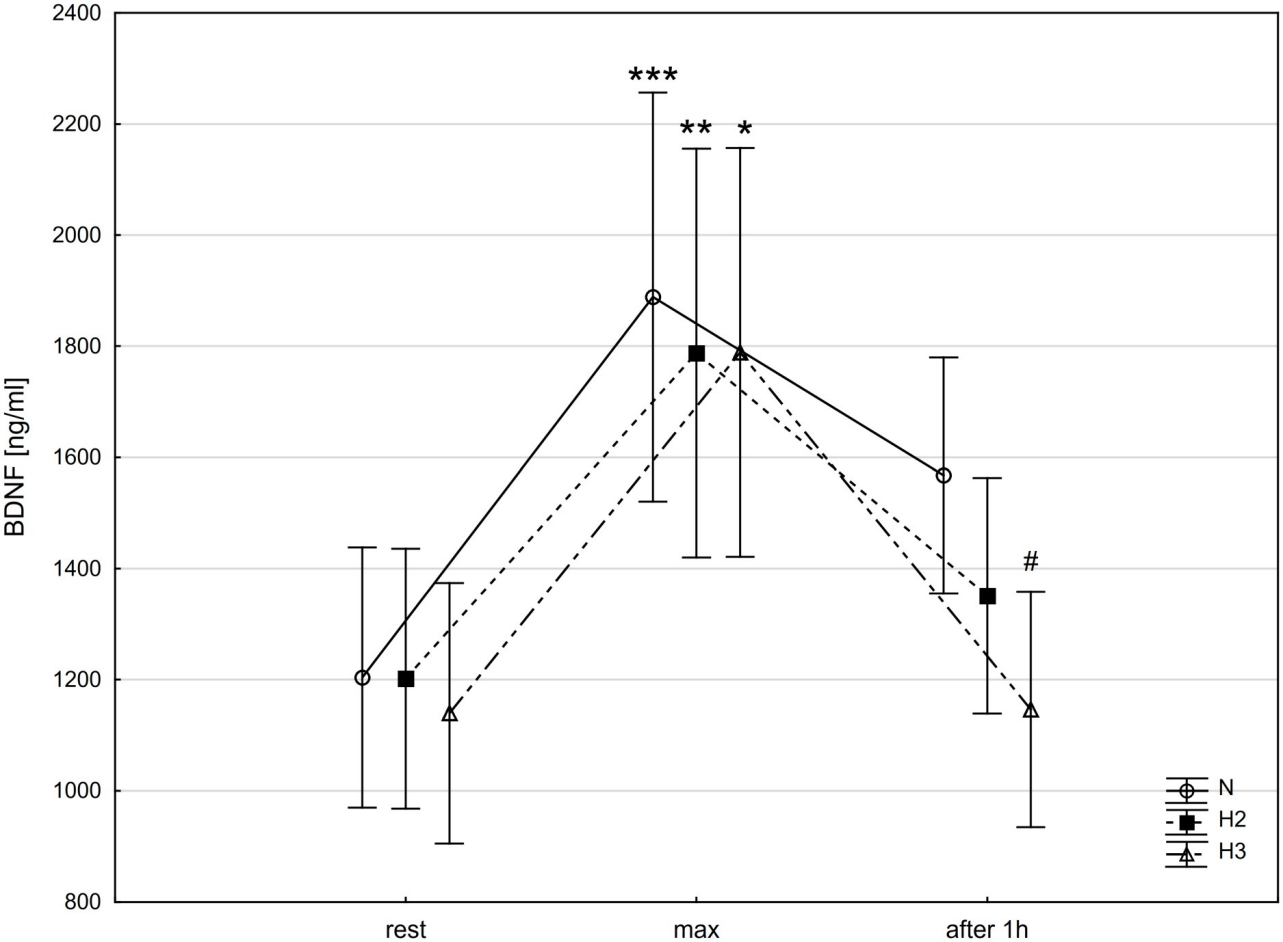
Brain-derived neurotrophic factor (BDNF) at rest, immediately after incremental test (max) and after 1 h recovery period in different conditions. N-normoxia, H2-hypoxia 2,000 m, H3-hypoxia 3,000 m; * p<0.05, ** p<0.01, *** p<0.001—statistically significant differences in relation to rest; # p<0.05—statistically significant differences in relation to max.

The post-hoc Tukey's test showed that BDNF concentration increased significantly (p<0.01) immediately after the incremental test (BDNF_max_) in N (by 56.8%; ES: 2.23), as well as in H2 (by 48.7%; ES: 1.80) and H3 (by 56.9%; ES: 1.0). Additionally, BDNF concentration decreased significantly (p<0.05) after a 1 h recovery period (BDNF_rest_) by 35.6% (ES: 1.18) only in the H3 trial (Fig 4).

**Fig 5:**
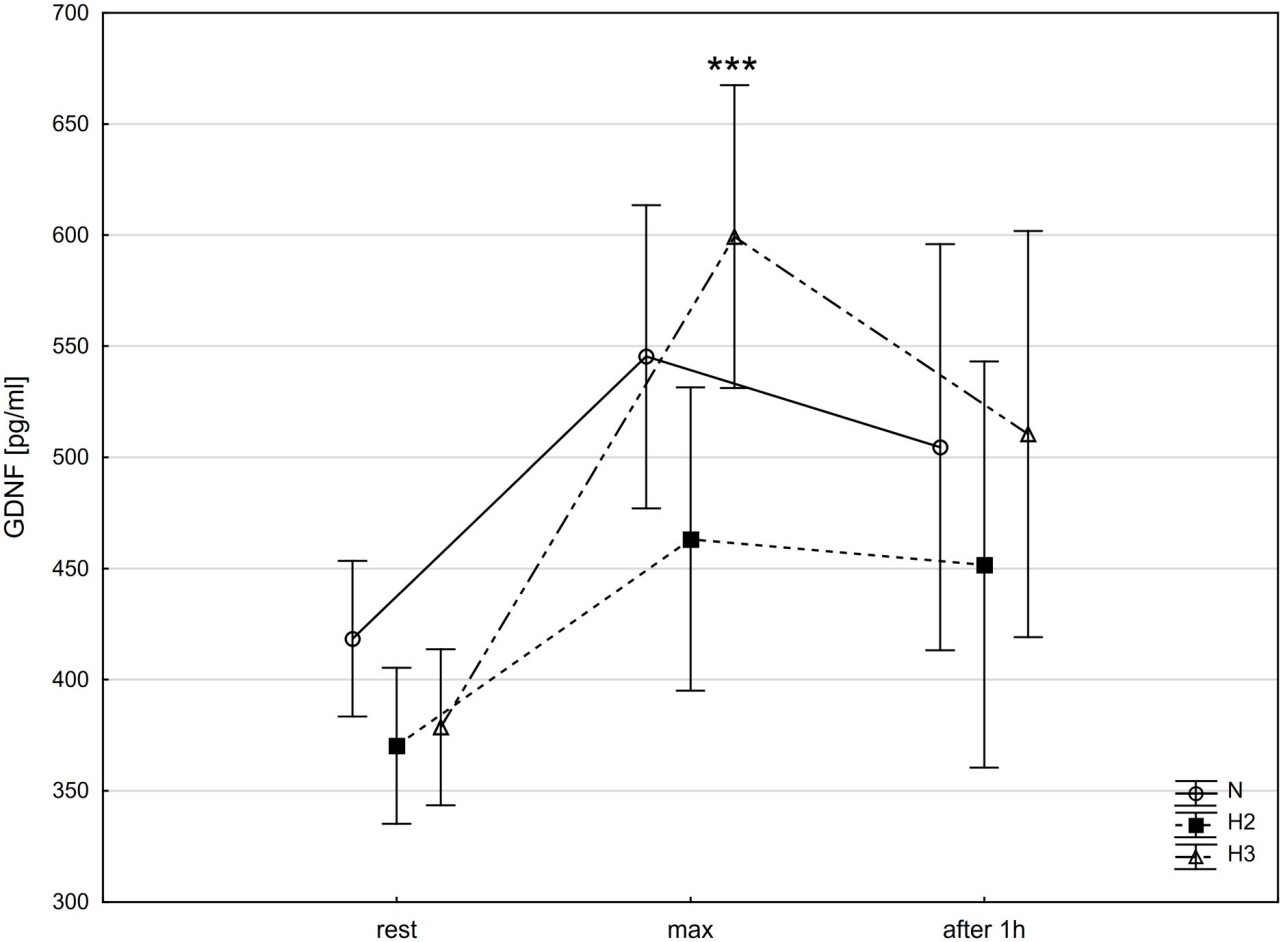
Glial cell line-derived neurotrophic factor (GDNF) at rest, immediately after incremental test (max) and after 1 h recovery period in different conditions. N-normoxia, H2-hypoxia 2,000 m, H3-hypoxia 3,000 m; *** p<0.001—statistically significant differences in relation to rest.

Similar changes were observed in GDNF concentration. The post-hoc Tukey's test showed that GDNF concentration increased significantly (p<0.01) immediately after the incremental test (GDNF_max_) by 58.2% (ES: 2.75), but only in H3 (Fig 5).

### Dopamine and metabolites changes

There was no significant interaction (condition x time of measure) effect on DA and its metabolites’ serum levels, but there was a significant effect of time of measurement (at rest, immediately after the test and 1 h recovery period) on DA (F = 15.26; p<0,0001) and DOPAC (F = 13.89; p<0.0001) concentration. Moreover, there was a significant effect of condition (N, H2, H3) on DA (F = 7.66; p<0.01) concentration and time of measurement on DOPAC/DA (F = 3.94; p<0.05) ratio. There was no significant effect of time of measurement or condition on HVA level or HVA/DA ratio.

The post-hoc Tukey's test showed that immediately after the incremental test serum concentration of DA increased significantly (p<0.05) by 41.9% but only in H2 and level of DOPAC increased significantly (p<0.01) by 224.7% in H3 (Table 2).

**Table 2:** Mean values of dopamine and selected metabolites measured in the different conditions at rest, after incremental test (max) and after 1 h recovery period.

Variables	N			H2			H3		
	rest	Max	after 1 h	rest	max	after 1 h	rest	max	after 1 h
**DA** [pg/l]	6.8±1.8	10.5±3.3	10.6±1.8	**10.5±2.6**	14.9±3.7*	11.2 ±2.1	10.6±2.9	14.7± 2,.8	12.6±1.7
**DOPAC** [pg/l]	20.4±6.1	37.3±10.7	33.5±20.0	24.7±11.0	49.8±33.3	28.7±19.8	**19.8±10.4**	64.3±25.3**	50.8±15.2
**HVA** [pg/l]	6.6±4.2	6.1±4.5	6.8±3.5	4.2±2.1	9.4±14.4	8.9±7.5	2.6±1.7	3.6±2.7	4.1±2.3
**DOPAC/DA**	3.12±0.88	3.75±1.30	3.22±2.05	2.34±0.74	3.88±3.51	2.52±1.58	2.04±1.16	4.35±1.61	3.99±1.05
**HVA/DA**	1.16±1.02	0.60±0.53	0.62±0.29	0.45±0.29	0.82±1.52	0.81±0.63	0.29±0.24	0.27±0.25	0.34±0.21

N- normoxia, H2—hypoxia 2,000 m, H3—hypoxia 3,000 m, DA—dopamine, DOPAC— 3,4-dihydroxyphenylacetic acid, HVA—homovanillic acid;

* p<0.05,

**p<0.01 statistically significant differences in relation to rest.

### Serotonin and metabolites changes

There was a significant interaction (condition x time of measure) effect in the 5-HT (F = 2.76; p<0.05) and in 5-HIAA (F = 5.14; p<0.01) serum concentrations, and a significant main effect of time of measurement but only in 5-HT concentration (F = 16.89; p<0.00001).

There was no significant effect of time of measurement or condition or interaction (condition x time of measure) on 5-HIAA/5-HT ratio.

The post-hoc Tukey's test showed a significant increase in serum 5-HT concentration immediately after the incremental test by 152.4% in N (p<0.01) and in H3 by 157.8% (p<0.05). Additionally, in H3 after 1 h of recovery serum concentration of 5-HT was higher than the resting value (by 178.9%; p<0.01).

The post-hoc Tukey's test showed significant differences in 5-HIAA concentration after 1 h of recovery between N and H3 (in H3 was higher by 157.4%; p<0.05) and H2 and H3 (in H3 it was higher by 215.49%; p<0.05). Moreover, after 1 h of recovery in H3 the level of 5-HIAA was higher by 379.3% compared to resting concentration (p<0.05, Table 3).

**Table 3:** Mean values of serotonin and selected metabolites measured in the different conditions at rest, after incremental test (max) and after 1 h recovery period.

Variables	N			H2			H3		
	rest	max	after 1 h	rest	max	after 1 h	rest	max	after 1 h
**5-HT** [pg/l]	**114. 3±37. 2**	288. 5±142. 8**	169. 2±47. 1	176. 4±67. 0	271. 7±116. 8	189. 4±68. 9	**103. 9±39. 8**	267. 9±99. 3*	**290. 7±155. 8**##
**5-HIAA** [pg/l]	11. 3±4. 8	2. 9±1. 7	**5. 4±4. 5**	5. 2±5. 0	3. 2±2. 3	**4. 4±3. 3**	**2. 9±3. 1**	4. 8±3. 0	**13. 9±13. 1**#&^
**5-HIAA** **/5HT**	0. 11±0. 05	0. 02±0. 02	0. 04±0. 04	0. 03±0. 03	0. 01±0. 01	0. 02±0. 02	0. 03±0. 02	0. 02±0. 02	0. 14±0. 30

N- normoxia, H2—hypoxia 2,000 m, H3—hypoxia 3,000 m, 5-HT— 5-hydroxytryptamine (serotonin), 5-HIAA— 5-hydroxyindoleacetic acid;

*p<0.05,

** p<0.01 statistically significant differences in relation to rest;

^#^p<0.05,

^##^p<0.01 statistically significant differences in relation to rest;

^&^p<0.05 statistically significant differences in relation to N after 1h;

^ p<0.05 statistically significant differences in relation to H2 after 1h

## Discussion

In agreement with previous studies that showed predominantly acute aerobic exercises as a potent stimulus for increasing blood-borne BDNF in humans, in the present study augmented BDNF elevation in blood serum was seen in response to EVE involving large muscle groups. This mode of exercise, especially at higher loads, contains a large muscle strength component. Assuming in accordance with the current state of knowledge, that basal and post-exercises serum BDNF levels are 70–80% of cerebral origin [61–62], our results revealed that this type of exercise that is metabolically distant from endurance exercise [63] is also effective at increasing circulating BDNF of brain origin. Skeletal muscles can also produce BDNF. It needs to be highlighted that muscle originating BDNF is not released into the circulation but acts in muscle in an autocrine and/or paracrine [64]. Previous research demonstrates that higher intensity exercise, compared to lower intensity exercise, may be more effective in augmenting neurotrophic levels [65,66], which is in line with our observation. It suggests that the BDNF-related advantageous effect of exercise on the brain depends on its characteristics, e.g. intensity and duration, being more effective at higher values of these variables [13]. In the present study, performed in conditions of moderate hypoxia, the EVE did not affect release of BDNF. This is in contradiction to a study which also involved non-trained men yet applied exercises with lower relative intensity (50% VO_2_max), longer duration and performed at more severe hypoxia (about 4,500 m altitude), which induced symptoms of AMS [67]. Moreover, the results from a recent study indicated that repeated daily intermittent normobaric hypoxia reduces BDNF plasma levels in young adults [68]. The basis of these differences has yet to be clearly elucidated.

The aforementioned data suggest that the exercise-induced changes in BDNF depend on multiple factors: the time of exposure, altitude levels, and the intensity of the exercise. In line with this inference are ambiguous human studies that investigated the effects of exposure to hypoxia on BDNF expression in resting conditions. The discrepancies concerning different response to acute hypoxia are most likely due to different exposure time and/or environmental oxygen saturation [69–71]. In our study, each participant remained in hypoxia for 0.5 h before performing the incremental test, and after this time, we did not observe significant changes in BDNF concentration compared to normoxia. Interestingly, there are data showing that 0.5 h exposure to hypoxia caused elevated serum BDNF levels compared to normoxia in higher hypoxic conditions (SpO_2_ = 75%) [69]. Some authors suggest, as the main mechanism of inducing changes in the CNS under oxygen depletion, involvement of hypoxia-inducible factors (HIF) [72], which may play an important role in the proliferation and differentiation of neurons [73]. However, a high dose of intermittent hypoxia (4,000–5,000 m of altitude 12x5 min/day for two weeks) led to a decrease in BDNF levels in serum [68]. Moreover, it was suggested that the effect on BDNF formation shows a biphasic pattern: the range of hypoxia of about 9–16% in inspired O_2_ had a positive effect while 2–8% inspired O_2_ had a negative effect [48]. Because the oxygen content in our study was in the range to elevate basal BDNF levels by 16.6% (H2) and 14.7% (H3), the lack of such changes suggests that pre-exercise exposure to hypoxia was too short.

In human acute hypoxia causes a reduction in VO_2_max [74]. However it should be mentioned that appropriate exposure to hypoxic condition (i.e. having a suitable exposure time and oxygen saturation/altitude level) can lead to an increase in VO_2_max in normoxia by inducing adaptations at physiological, biochemical and genetic levels [75] especially if it is combined with a properly selected intensity of training [76,77]. Reduction in VO_2_max occurred in our subjects performing physical effort in conditions of normobaric hypoxia and was, as reported by others, directly related to the degree of hypoxia. Despite the fact that achieved absolute work intensity was lower in hypoxia, the plasma BDNF levels were similar to those seen in normoxia. These observations show that changes in metabolism at the skeletal muscle level, e.g. skeletal muscle O_2_-limited ATP regeneration during acute hypoxia with an increased rate of glycolysis, do not meaningfully affect brain BDNF production [78]. The similarity of the BDNF concentrations in all trials applied in our study implies that exercise intensity is a superior and stronger factor for stimulation of BDNF formation. It was observed that at maximal exercise in hypoxia, the blood LA concentration was at least similar to or even higher than that during normoxia [79]. A similar tendency occurred in the present study, e.g. LA concentrations were higher in response to maximal effort in both hypoxic conditions. These observations lead to the conclusion that systemic LA production does not stimulate BDNF response to maximal exercise. The last effect was recently demonstrated in response to maximal resistance exercise [66].

It is well established that GDNF is highly expressed during development, especially in the stratum, but it has also been found in adult brain, yet in lower concentrations. In the present study, EVE caused an elevation of circulating GDNF similar to that observed in BDNF. Therefore, it is reasonable to hypothesize that this type of exercise stimulates both neurons and astrocytes in parallel. While in the case of stimulation of BDNF production in response to exercise may be explained by sensory nerve impulses originating from working muscles, the astrocytes’ stimulation for GDNF production may be attributed to discharging chemical messengers from muscles into the circulation [80] and translocated to the CNS. Among likely potent messengers that can communicate skeletal muscle activity during maximal exercise to the brain is metabolically generated ammonia (mostly present as ammonium—NH_4_^+^) produced in fast twitch fibers [81,82]. Ammonia alters astroglial metabolism, influences signaling transduction pathways, activities many enzymes and generates free radicals [83,84]. In response to elevated serum NH_4_^+^ glutamine content in the CNS rises though glutamine synthase activity increases, causing astrocyte to swell. Another candidate is muscle produced interleukin-6 (IL-6), which can be transported to the brain in an amount to produce biological effects [85]. Circulating IL-6 increases markedly during strenuous exercise as a result of muscle contraction and a low muscle glycogen content [86,87]. It was reported that IL-6 was also produced by activated astrocytes [88] and could be released in humans from the brain to the circulatory system [89]. However, this release is many times smaller than the release of IL-6 from exercising muscles [89]. Moreover, this phenomenon was selectively documented in response to prolonged exercise at 50% of VO_2_max [89]. Unfortunately, no information is available from studies dealing with high intensity exercise that has been applied in the present study.

In face of the facts that GDNF exerts a pivotal role in the development and maintenance of spinal motor neurons and midbrain dopaminergic neurons [90], and BDNF triggers the transcription of the TH gene responsible for the conversion of tyrosine to L-DOPA, which is precursor of DA [24,25], and serotonergic and dopaminergic systems are tightly linked and influence each other [91], we also measured blood levels of DA and 5-HT in all examined conditions. These neurotransmitters are related to the “central fatigue hypothesis”. Although their contribution to central fatigue in humans is still poorly understood, it is postulated that augmented activity of the dopaminergic system is caused by the exercise-induced activation of TH by the calcium-calmodulin system [92], whereas the similar decrease in DA concentration seen during exercise results from the inhibitory effect of 5-HT [30]. Because our results revealed similar changes in circulating levels of BDNF and GDNF as well as DA and 5-HT in all investigated circumstances, while the absolute load at maximal exercises differed, it implied that these neurotrophins were probably stronger stimuli than exercise involved in their production. However, this hypothesis-generating assumption needs to be further investigated. Moreover, it is known that aerobic training caused a decrease in 5-HT levels immediately after exercise and 48h after its completion, which suggests that endurance training, in addition to metabolic [93] and peripheral tissues adaptation, can also lead to adaptive changes in the brain [94]. Therefore, in the future, we would also like to analyze the aspect of endurance training in the context of nerve cell activation and release of neurotrophins as well as the possible impact of strength training.

The consequences of too rapid ascent to altitudes might cause some pathophysiological effects on the brain, especially in so-called responders, among which the best recognized are high-altitude headache, AMS, high-altitude pulmonary edema and high-altitude cerebral edema. Some of these high-altitude illnesses are associated with rapid reduction in the driving oxygen after sudden exposure to hypobaric hypoxia [49]. In agreement with the supposition that the latter phenomenon occurred in our study during subjects’ exposure to normobaric hypoxia, we examined the state of the brain by means of measurement of selected neurotrophins which have been considered as markers of CNS damage, i.e. bFGF, S100B, GFAP and NGF [95–97]. These variables are most commonly used to control physiopathology of the brain. According to data from our study that revealed no changes in plasma levels of these factors, one can assume that neither exercise to volitional exhaustion nor hypoxia nor their combination causes deterioration of CNS function. In line with such an assumption are comments from our participants who did not complain of some harmful effects, e.g. headache, nausea or stupor, which could develop even during acute short-term exposure to moderate hypoxia. However, this conclusion follows from hypothesis-generating evidence. This is because the majority of the literature on this subject involves elevation of serum neurotrophins in patients who have traumatic brain injury [98]. There is also a study showing that elevated plasma S1000B in high altitude hypobaric hypoxia does not correlate with AMS [99]. In addition, increased expression of bFGF in astrocytes is observed in brain damage and ischemia, suggesting that it may be a protective factor to save neurons [96]. The question arises how the level of bFGF in the serum reflects the level of its expression in the brain. According to the authors' knowledge, there are no data that allow one to compare bFGF expression in the brain and its levels in serum. However, according to a study that showed no changes in bFGF distribution in muscles after physical training and the fact that bFGF was located primarily in the cytosol, sarcolemma and intercellular space of myocytes, but not in smooth muscle cells of the blood vessels, we believe in the high release of bFGF from the CNS [100].

Calcium binding protein B and GFAP are mainly produced by astrocytes and are widely considered as markers of brain injury [101,102]. It is assumed that S100B is one of the peripheral markers of activation of glial cells [103] in response to brain damage. This phenomenon can also occur during physical exercises, in which the athlete's body and or head is exposed to body strikes or punches (e.g. boxing, karate, but also team games) [104]. Importantly, it was shown that running (both sprint and long-distance running) leads to an increase in S100B in human serum, but after the maximal exercise performed on a cycle ergometer there was no such effect [105]. The latter observation was attributed to lack of changes in BBB permeability that can be changed by running-induced vibrations. Hence, there is no increased translocation of S100B from the CNS to the bloodstream [105]. Concentrations of S100B measured in our individuals were relatively low in all experimental conditions (less than 0.015 ng/ml), but it is assumed that higher values (0.3 ng/ml in patients with schizophrenia) are neurotoxic [104]. It indicates that S100B can be either a neurotropic or a neurotoxic factor depending on its concentration in the circulation. Again, all abovementioned results and the fact that maximal exercise applied in our study did not elevate serum levels of these neuropeptides under any examined conditions suggest that all these experimental condition do not induce deterioration of CNS function.

The impact of exercise on GFAP expression is ambiguous. It was reported an increase S100B without an increase in GFAP in serum, during the same exercise protocol, although both of these neurotrophins are mainly of astrocyte origin [106,107]. Our study showed that exercise did not cause significant changes in release of GFAP, which is consistent with the reports cited above and again in accordance with the assumption that our individuals exercised with no impairment of brain function.

It is well established that NGF is produced by neurons, astrocytes, pericytes, immune cells and the pituitary gland [108]. Therefore, it is problematic to evidence the source of NGF origin in the blood. Increased values of NGF were observed under mental stress in the circulatory system and the source was probably immune cells [109]. It is also known that under severe hypoxia there was an increase in NGF concentration in nerve cells [110]. Moreover, hypoxia (1% O_2_) caused an increase in NGF production mainly by astrocytes in order to transfer NGF to neurons, because during hypoxia neurons have the greatest demand for trophic factors [97]. In our study, concentration of NGF in both hypoxic conditions was higher compared to normoxia, but this result is not statistically significant. It is possible that this phenomenon was due to the response from neurons and / or astrocytes to hypoxia. However, the level of hypoxia was insufficient to clearly induce the significant effect on the NGF level.

## Conclusion

The present study showed that maximal exercise to volitional exhaustion performed on a cycle ergometer simultaneously activates both neurons and astrocytes, which was reflected by elevated levels of BDNF and GDNF in the blood of healthy young sedentary men. The increased secretion of these neuropeptides was not the result of brain damage because the blood levels of other neurotrophins considered as markers of CNS disruption (i.e., s100B, GFAP, NGF, bFGF) did not change in any of the experimental conditions (normoxia and hypoxia). Thus, the obtained results also indicate that maximal exercise performed in moderate normobaric hypoxia (up to 3,000 m; FiO_2_ = 14.7%) is safe for the CNS of healthy young men.

## Limitations and outlook

One of the limitations of the study is small sample size (N = 7). This is due to the fact that during the recruitment of volunteers, after explained the possible risk of performing exercises under hypoxia condition, some candidates resigned from participation in the study. Next limitation is the lack of information about cognitive function before and after intervention. Future studies should include this aspect.

## Supporting information

S1 Table Raw data. (PDF)

## Abbreviations

5-HIAA: 5-hydroxyindoleacetic acid
5-HT: serotonin
AMS: acute mountain sickness
BBB: blood-brain barrier
BDNF: brain-derived neurotrophic factor
bFGF: basic fibroblast growth factor
CNS: central nervous system
CO_2_: expired carbon dioxide
DA: dopamine
DOPAC: 3,4-dihydroxyl phenylacetic acid
EVE: exercise test to volitional exhaustion
GDNF: glial cell line-derived neurotrophic factor
GFAP: glial fibrillary acidic protein
H2: hypoxic condition equivalent 2,000m altitude
H3: hypoxic condition equivalent 3,000m altitude
HVA: homovanillic acid
IL-6: interleukin-6
LA: lactate
N: normoxic condition
NGF: nerve growth factor
S100B: calcium binding protein B
VO_2max_: maximal oxygen uptake
WR_max_: maximum workload
