# Supplementary material for: Acute normobaric hypoxia does not affect the simultaneous exercise-induced increase in circulating BDNF and GDNF in young healthy men: A feasibility study
Source: PLoS One. 2019 Oct 23;14(10):e0224207. doi: 10.1371/journal.pone.0224207 (PMC6808427; doi:10.1371/journal.pone.0224207)
Supplement: S1 Table — (PDF) [file pone.0224207.s001.pdf]

Subjects	BDNF Nrest [ng/ml]	BDNF Nmax [ng/ml]	BDNF N1h [ng/ml]	BDNF H2rest [ng/ml]	BDNF H2max [ng/ml]	BDNF H2 1h [ng/ml]	BDNF H3rest [ng/ml]	BDNF H3max [ng/ml]	BDNF H3 1h [ng/ml]
1	1027	1425	1034	1304	1485	1099	972	1347	905
2	1033	1832	1288	1263	1623	1319	832	2597	1256
3	1299	1804	1468	1436	2230	1670	1638	2096	1021
4	1253	2738	1777	1532	2191	1430	1508	2118	1590
5	1412	2204	1746	1118	2076	1448	1562	1827	1280
6	1220	1646	1729	1019	1825	1443	1217	1217	1217
7	1183	1571	1930	740	1083	1047	774	692	840

Subjects	GDNF Nrest [pg/ml]	GDNF Nmax [pg/ml]	GDNF N1h [pg/ml]	GDNF H2rest [pg/ml]	GDNF H2max [pg/ml]	GDNF H2 1h [pg/ml]	GDNF H3rest [pg/ml]	GDNF H3max [pg/ml]	GDNF H3 1h [pg/ml]
1	463,16	721,05	463,16	336,84	426,32	684,21	347,37	563,15	505,98
2	378,95	584,21	405,6	331,58	357,89	312	497,37	705,26	633,87
3	431,48	510,53	531,58	347,37	468,42	403,3	357,89	447,37	447,76
4	400,43	542,11	768,42	367,98	498,89	399,76	315,79	621,05	498,87
5	378,95	468,42	457,89	401,21	498,76	399,76	373,68	478,37	398,98
6	426,32	485,42	373,68	432,96	518,42	568,42	400,01	653,63	588,76
7	449,74	505,26	531,58	373,68	473,68	394,74	357,89	726,32	498,76

Subjects	bFGF Nrest [pg/ml]	bFGF Nmax [pg/ml]	bFGF N1h [pg/ml]	bFGF H2rest [pg/ml]	bFGF H2max [pg/ml]	bFGF H21 h [pg/ml]	bFGF H3rest [pg/ml]	bFGF H3max [pg/ml]	bFGF H3 1h [pg/ml]
1	3,20	7,38	3,05	2,90	4,10	5,08	2,58	4,03	3,28
2	4,30	4,38	3,25	3,48	3,63	6,00	3,80	5,43	3,40
3	3,25	4,87	2,5125	4,65	0,93	0,83	6,05	3,85	0,50
4	3,78	5,10	2,51	4,68	3,00	1,65	1,38	9,58	6,10
5	3,22	5,02	2,43	3,08	3,45	2,45	6,95	6,75	4,95
6	3,33	3,98	1,08	1,18	0,95	6,33	2,88	3,80	5,63
7	1,48	4,68	2,68	3,78	7,20	5,75	4,10	3,30	3,20

Subjects	NGF Nrest [pg/ml]	NGF Nmax [pg/ml]	NGF N1h [pg/ml]	NGF H2rest [pg/ml]	NGF H2max [pg/ml]	NGF H2 1h [pg/ml]	NGF H3rest [pg/ml]	NGF H3max [pg/ml]	NGF H3 1h [pg/ml]
1	1307	903	935	6516	3710	15903	3581	1935	3677
2	1097	1548	3613	46645	38516	13742	21806	24742	22032
3	1257	1446	7032	1129	1355	2000	10226	12871	4839
4	2348	1392	3397	2129	2645	12097	2323	2419	7452
5	1406	1411	3235	30323	12065	1000	2452	3194	8806
6	648	1161	1065	1839	2097	1548	8806	7548	8968
7	1129	1581	1839	17129	58774	42290	1839	4355	4194

Subjects	S100B Nrest [pg/ml]	S100B Nmax [pg/ml]	S100B N1h [pg/ml]	S100B H2rest [pg/ml]	S100B H2max [pg/ml]	S100B H2 1h [pg/ml]	S100B H3rest [pg/ml]	S100B H3max [pg/ml]	S100B H3 1h [pg/ml]
1	6,1	2,6	10,6	11,00	14,8	8,2	6,4	14,6	8,6
2	14	0	0	38,6	0,4	3,2	22,6	43,6	2,4
3	5,8	3,9	9	1,8	2,4	10,8	17	36	27
4	0	3,3	6,8	12,8	5,4	11,8	26,4	0	4,6
5	7,2	2,9	7	4,4	13,4	15,8	16,8	17,6	23,2
6	2,2	4,4	3,6	17,4	14,8	6,8	48,2	19,8	23,6
7	10,2	7	11	14,60	11,4	44,4	26	14,4	16,4

Subjects	GFAP Nrest [pg/ml]	GFAP Nmax [pg/ml]	GFAP N1h [pg/ml]	GFAP H2rest [pg/ml]	GFAP H2max [pg/ml]	GFAP H2 1h [pg/ml]	GFAP H3rest [pg/ml]	GFAP H3max [pg/ml]	GFAP H3 1h [pg/ml]
1	1,04	1,37	0,85	2,75	6,59	1,10	2,10	1,81	3,48
2	1,25	1,87	0,92	0,64	1,38	1,77	2,24	1,24	1,54
3	0,74	1,55	0,74	40,43	0,74	14,82	1,17	1,84	0,75
4	5,49	1,63	0,71	40,43	39,09	3,84	7,13	2,95	1,06
5	1,86	1,97	0,93	1,21	1,24	2,04	1,19	1,93	1,52
6	0,73	0,96	0,74	0,74	11,35	1,96	2,24	1,05	1,62
7	2,21	2,60	0,89	5,68	1,18	0,85	1,20	1,74	4,48

Subjects	DA Nrest [pg/l]	DA Nmax [pg/l]	DA N1h [pg/l]	DA H2rest [pg/l]	DA H2max [pg/l]	DA H2 1h [pg/l]	DA H3rest [pg/l]	DA H3max [pg/l]	DA H3 1h [pg/l]
1	6,93	7,82	8,59	11,82	21,76	7,01	12,55	17,37	11,67
2	8,28	9,32	8,74	12,94	14,83	11,86	14,06	15,67	13,71
3	7,32	11,13	10,4	12,67	13,21	12,05	5,39	17,83	12,36
4	6,78	10,63	12,44	5,89	17,02	12,32	11,82	15,67	15,56
5	9,01	17,56	13,17	11,13	14,63	13,52	11,32	13,71	13,4
6	3,35	8,39	9,42	8,25	9,74	10,91	8,25	9,74	10,91
7	5,91	8,87	11,73	10,72	13,21	10,73	10,72	13,21	10,73

Subjects	DOPAC Nrest [pg/l]	DOPAC Nmax [pg/l]	DOPAC N1h [pg/l]	DOPAC H2rest [pg/l]	DOPAC H2max [pg/l]	DOPAC H2 1h [pg/l]	DOPAC H3rest [pg/l]	DOPAC H3max [pg/l]	DOPAC H3 1h [pg/l]
1	14,95	40,55	56,33	19,45	20,33	19,09	10,66	54,21	64,19
2	28,55	30,67	29,07	21,33	39,78	42,36	40,45	56,02	67,55
3	24,52	32,43	36	41,07	82,5	43,55	21,31	95,69	53,22
4	26,9	59,9	62,07	13,5	56,79	57,21	8,22	68,69	57,83
5	17,84	35,07	28,09	38,17	24,62	26,69	17,84	44,95	50,74
6	14,38	34,45	6,26	14,33	104,9	7,19	20,53	31,29	24,67
7	15,72	27,98	16,91	25,24	19,86	4,86	19,81	99,1	37,09

Subjects	HVA Nrest [pg/l]	HVA Nmax [pg/l]	HVA N1h [pg/l]	HVA H2rest [pg/l]	HVA H2max [pg/l]	HVA H2 1h [pg/l]	HVA H3rest [pg/l]	HVA H3max [pg/l]	HVA H3 1h [pg/l]
1	5,47	4,41	4,11	3,88	8,58	6,79	2,77	5,47	2,57
2	1,84	1,65	1,51	1,17	1,2	1,52	1,37	0,56	5,03
3	0,53	3,6	3,97	7,26	2,35	22,74	3,55	1,12	1,16
4	6,9	6,15	9,08	5,39	5,5	4,05	1,2	4,08	4,8
5	9,64	9,05	9,55	3,55	2,88	9,27	0,53	0,87	2,01
6	10,06	14,78	10,01	5,77	41,53	14,43	3,24	7,15	5,66
7	11,45	2,79	9,12	2,16	3,93	3,42	5,63	5,66	7,54

Subjects	5-HT Nrest [pg/l]	5-HT Nmax [pg/l]	5-HT N1h [pg/l]	5-HT H2rest [pg/l]	5-HT H2max [pg/l]	5-HT H2 1h [pg/l]	5-HT H3rest [pg/l]	5-HT H3max [pg/l]	5-HT H3 1h [pg/l]
1	126,56	232,17	227,97	141,36	173,67	169,64	63,92	437,04	312,67
2	154,66	314,11	191,98	173,61	330,81	195,26	82,92	161,8	363,92
3	165,26	393,28	157,43	152,13	203,61	130,19	140,5	247,02	262,57
4	114,41	182,48	120,81	103,93	153,17	110,73	81,19	331,09	312,67
5	77,45	242,36	135,14	151,27	232,28	196,01	64,49	222,26	44,91
6	67,66	540,98	122,65	313,24	485,99	324,47	159,5	312,09	550,48
7	94,15	114,01	228,6	199,23	322,46	199,23	135,03	163,82	187,43

Subjects	5-HIAA Nrest [pg/l]	5-HIAA Nmax [pg/l]	5-HIAA N1h [pg/l]	5-HIAA H2rest [pg/l]	5-HIAA H2max [pg/l]	5-HIAA H2 1h [pg/l]	5-HIAA H3rest [pg/l]	5-HIAA H3max [pg/l]	5-HIAA H3 1h [pg/l]
1	20,39	4,44	3,32	3,11	0,47	2,5	1,29	4,73	4,05
2	4,97	1,44	3,73	1,76	1,18	1,53	2	2,56	22,68
3	8,55	1,47	3,61	3,17	1,7	3,47	2,88	5,32	3
4	12,93	1,67	3,47	5,38	3,23	2,62	2,23	4,03	21,77
5	9,43	3,03	3,56	16,16	4,05	3,88	1,38	6,61	36,46
6	11,78		15,51	4,14	6,93	5,32	0,91	0,44	3,82
7	11,08	5,47	4,58	2,82	4,7	11,22	9,73	9,73	5,73

Subjects	ΔLA N	ΔLA H2	ΔLA H3	Wrmax N	Wrmax H2	Wrmax H3	VO2max N	VO2max H2	VO2max H3
1	10,93	11,82	10,67	320	280	253	56	49	49
2	14,91	15,51	17,01	280	280	260	58	49	45
3	10,08	10,2	11,62	320	280	270	55	46	40
4	13,62	12	13,35	280	240	220	53	43	42
5	9,05	9,47	9,73	320	280	260	53	50	47
6	8,11	9,21	9,98	240	226	200	42	39	33
7	9,42	10,11	10,95	260	226	213	49	39	36
